# Physical inactivity in early pregnancy and the determinants in an urban city setting of Kuala Lumpur, Malaysia

**DOI:** 10.1186/s12889-022-12513-5

**Published:** 2022-01-13

**Authors:** Sharifah Fazlinda Syed Nor, Idayu Badilla Idris, Zaleha Md Isa

**Affiliations:** 1 Department of Community Health, Faculty of Medicine, Universiti Kebangsaan Malaysia, Jalan Yaacob Latif, Bandar Tun Razak, Batu 9 Cheras, 56000 Wilayah Persekutuan Kuala Lumpur, Malaysia; 2 Training Management Division, Ministry of Health Malaysia, Kompleks E, Pusat Pentadbiran Kerajaan Persekutuan, 62590 Wilayah Persekutuan Putrajaya, Malaysia

**Keywords:** Exercise, Pregnant women, Factors, First trimester, PPAQ

## Abstract

**Background:**

Physical inactivity in pregnancy has been associated with excessive gestational weight gain, hypertensive disorders, gestational diabetes mellitus and postpartum depression. Despite these risks, physical inactivity level remains high especially in higher income countries. The prevalence of physical inactivity among women in Malaysia aged ≥16 years was 28.2% in 2019 exceeding men by 6.1%. However, little is known regarding the subpopulation of pregnant women especially in Kuala Lumpur which is the most urbanized and highly populated city in Malaysia. Therefore, the aim of this study is to measure the physical inactivity prevalence among first trimester pregnant women in Kuala Lumpur and to identify its determining factors.

**Methods:**

This was a cross-sectional study in which 339 first trimester pregnant women were sampled from 13 maternal and child health clinics located in all four parliament districts of Kuala Lumpur. Self-administered questionnaires which contained the Malay version of the pregnancy physical activity questionnaire (PPAQ) were used. Descriptive analysis was conducted to determine the physical inactivity prevalence followed by simple and multiple logistic regression to identify the determinants of physical inactivity with significant level of 5%.

**Results:**

The prevalence of physical inactivity was 38.3%. The highest activity was seen in the household activity domain, despite only 24.8% of the respondents were housewives/unemployed. There was little to no participation observed in the vigorous intensity category. The determinants of physical inactivity were primigravida (aOR 3.54 95% CI 1.40, 8.97), education level (aOR 3.77 95% CI 1.35, 10.52) and body mass index (aOR 0.88 95% CI 0.80, 0.97) which explained 22.6% variation of physical inactivity in the final adjusted model.

**Conclusion:**

The prevalence of physical inactivity among first trimester pregnant mothers in this study was 38.3%, and the highest activity was seen in the household category. Health education on physical activity in pregnancy should be focused on those who are primigravida and have no tertiary education. The educational content should be updated and tailored to current pandemic situation where self-isolation is the new norm, by advocating for home-based, moderate to vigorous intensity physical activities.

## Background

Physical activity can be defined as any movement produced by skeletal muscles that requires the use of energy [1]. It can be measured in metabolic equivalents (METs) [2]. As a reference, one metabolic equivalent of task (MET) equals the energy being used for sitting at rest [2]. The World Health Organization (WHO) recommends healthy adults to be involved in at least 150 min of moderate- to vigorous-intensity activity, which are activities of at least 3 METs, spread throughout the week [1]. This also applies to pregnant women because physical activity early in the first trimester and throughout the pregnancy [3] is not associated with increased odds for miscarriage or prenatal mortality [4]. Instead, it is strongly associated with reduced risks of excessive gestational weight gain, hypertensive disorders, gestational diabetes mellitus and postpartum depression [5–9].

Physical inactivity has been identified as one of the modifiable risk factors for non-communicable diseases in the general adult population; these diseases include cardiovascular disease, diabetes mellitus as well as certain cancers, among others [1]. It is also associated with mental health disorders such as depression [1]. Besides these morbidities, physical inactivity has also been identified as the fourth leading risk factor for mortality [10]. Global data have shown that physical inactivity is two times more prevalent in higher income countries compared with their low income counterparts, a phenomenon mostly attributed to rapid urbanisation, especially in the transportation network [11]. This phenomenon highlights the need for a more concerted effort to empower and promote physical activities and an active lifestyle among populations in developing and developed nations and cities around the world.

The overall global physical inactivity prevalence stood at 27.5% in 2016. It is worth noting that at 31.7%, the prevalence of physical inactivity in women exceeded the prevalence in men by 8.0%. Latin America, the Caribbean and South Asia had the highest prevalence of physically inactive women [12]. During pregnancy, the prevalence of physical inactivity tends to be higher than the general female population because pregnant women tend to reduce their physical activity once they become pregnant [13–16]. Recent studies have reported that physical inactivity in pregnant women ranged from 21.9% to as high as 75.0% [15, 17–21]. The highest physical inactivity prevalence was seen among pregnant women in Oslo, Norway, and the lowest was seen among pregnant women in Tigray, Ethiopia [17, 19]. However, it is worth noting that different physical inactivity measurement methods and outcomes were applied in these studies. Similar to the global data of the general population, a lower prevalence of physical inactivity among pregnant women was found in lower income countries.

The overall prevalence of physical inactivity among the Malaysian population aged ≥16 years was 25.1% in 2019, down sharply from 33.5% in 2015, as reported in the National Health and Morbidity Survey (NHMS) 2019 [22]. The criteria used in this survey to classify a person as physically active were similar to the criteria recommended by the WHO. Specifically, the criteria were people involved in at least 20 min of vigorous activity for three or more days in a week or 30 min of moderate activity for five or more days in a week or achieving a total of at least 600 MET-minutes of activity in a week [22]. The NHMS 2019 also revealed that physical inactivity was more prevalent in the urban populations and similar to the global data. Moreover, the prevalence of physical inactivity was higher among women (28.2%) compared with men [22]. Despite these findings, the latest study by Su et al. [23] found only 17.3% of women in the low-income category in Kuala Lumpur were physically inactive.

There are no national data regarding physical inactivity specifically during pregnancy in Malaysia. However, the prevalence of physical inactivity is expected to be much higher in pregnant compared with non-pregnant women because women generally tend to reduce their physical activity once they become pregnant and as the pregnancy progresses [13, 15, 16, 24]. It is also worth noting that physical activity in the first trimester may have the most positive impact on the well-being of the pregnancy as a whole, because not getting sufficient physical activity from the first trimester is highly associated with pregnancy complications such as gestational diabetes and hypertensive disorders [3, 25]. In addition, early initiation of physical activity in the first trimester has been linked to improved and greater sleep quality [26]. Although first trimester participation in sports and exercise was found to reduce the infant’s birth weight, it did not increase the risk of delivering small for gestational age babies [27]. Therefore, early detection and identification of physical inactivity among pregnant women is vital so that early and timely interventions can be delivered to prevent pregnancy complications related to physical inactivity.

Individual factors that have commonly been associated with physical activity in pregnancy are parity or number of living children, education level, employment status and body mass index (BMI); maternal age, ethnicity and history of miscarriage have been considered less frequently [15, 17, 19, 20, 28–33]. Three studies have found that primiparity is significantly associated with physical inactivity [17, 19, 30], while three other studies have found that multiparity is significantly associated with physical inactivity [15, 31, 33]. However, the measurement tools and outcomes of these studies were not the same and had not been standardised, perhaps explaining the contradictory findings.

Gebregziabher et al. [17] reported that those who had lower education levels were likely to be physically inactive compared with those who went to high school (aOR 19.4 95% CI 4.9, 76.1). Similarly, a study in Serbia found that those with less than 12 years of formal education are twice as likely to be physically inactive compared with those who had more than 12 years of formal education (aOR 2.30 95% CI 1.05, 5.04) [20]. Meanwhile, Nascimento at al [15]. found that those who went to college or a higher institution are three times more likely to be involved in physical activity during pregnancy (aOR 3.0, 95% CI 2.0–4.5). However, two studies from Nigeria and Norway reported that education level is not significantly associated with physical activity levels [19, 31].

Previous findings regarding the association between BMI and physical activity levels have been inconclusive. However, most have found that higher BMI contributes to greater physical inactivity [19, 33, 34]. On the other hand, Santo et al. [35] found that underweight has an association with sufficient physical activity compared with a normal BMI. Studies from Brazil, Nigeria and Serbia have found no association between BMI and physical activity levels in pregnancy [15, 20, 31], although Oyeyemi et al. [31] from Nigeria reported that the waist-to-hip ratio is a significant factor to predict a sufficient physical activity level.

Another factor that has been found to be associated with physical activity levels is the employment status or maternal occupation. Gebregziabher et al. [17] reported that farmers in Ethiopia were more likely to be associated with sufficient activity level compared with housewives. Oyeyemi et al. [31] found that businesswomen in Nigeria were more likely to be physically active. Nascimento et al. [15] found that those who were working were more likely to be physically active compared with those who were unemployed. Three other studies from Sweden, Norway and Serbia, however, did not find any association between employment status and the physical activity level [19, 20, 34].

Realizing the importance of reducing the risks of physical inactivity in pregnancy, this study was conducted with the aim to measure physical activity levels and determine the prevalence as well as the factors associated with physical inactivity among first trimester pregnant women in an urban city setting of Kuala Lumpur, Malaysia.

## Methods

### Study design and background

This cross-sectional study was conducted among the population of pregnant women in the Malaysian capital, the Federal Territory of Kuala Lumpur. This area was selected because it is 100% urbanised and the risk factors for a high prevalence of physical inactivity are present in the population [11, 12, 22]. This city comprises four distinct parliament districts, namely Cheras, Kepong, Titiwangsa and Lembah Pantai. An estimated 24,235 pregnant women resided in Kuala Lumpur in 2019, of whom 28.9% were in Kepong, 18.0% in Lembah Pantai, 32.6% in Titiwangsa and 20.5% in Cheras.

### Sampling and data collection

We calculated the sample size by using the Power and Sample Size Program (PS version 3.1.2) software based on significant variables (parity, occupation, education level, BMI, history of miscarriage) from similar studies among pregnant women [15, 17, 35], with 80% power and an alpha value of 0.05 (95% CI). The highest sample size calculated was 282; with the addition of 10% to anticipate non-response, the final required sample size was 310. The respondents were conveniently sampled from 13 out of 24 government maternal and child health (MCH) clinics in all four parliament districts of Kuala Lumpur on their first day of antenatal check-up (registration/booking day).

The inclusion criteria were Malaysian citizens aged ≥18 years, first trimester or less than 13 weeks along in gestation (calculated from the date of last menstruation confirmed by ultrasound scan at booking), singleton pregnancy and able to read and speak in Malay or English. The exclusion criteria were multiple gestations (confirmed by ultrasound scan at booking), current medical illness (cardiovascular diseases, diabetes mellitus, anaemia, asthma etc.), physically or mentally disabled and > 40 years old. The recommendation to be physically active by the WHO – 150 min of moderate to vigorous activities in a week – only applies to healthy pregnant women [1], which was the reason why high risk pregnancies (women < 18 or > 40 years old, physically and mentally disabled, multiple gestation pregnancies) and those who had existing medical illnesses were not included in this study.

**Fig. 1:**
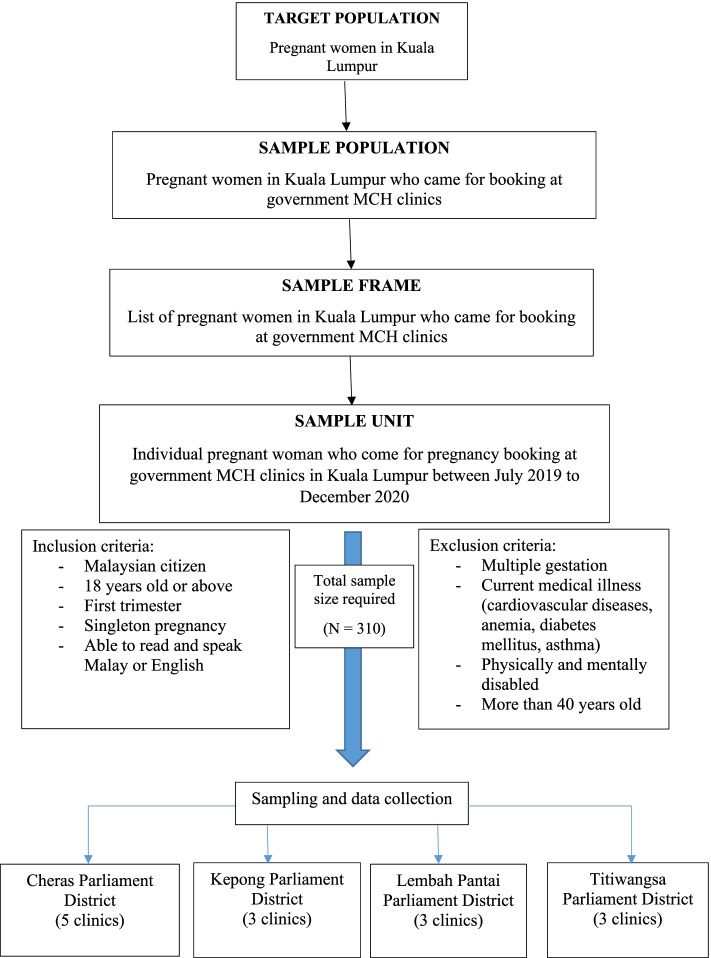
Flowchart of sampling

The study flowchart is presented in Fig. 1. The respondents voluntarily participated. Once written consent was obtained, participants were given a questionnaire to complete. Data collection was conducted from July 2019 to August 2020.

### Study instrument

This study used a self-administered questionnaire comprising five sections. Section A was on sociodemographic details, section B was on anthropometric details, section C was on medical history, section D was on 24-h dietary recall and section E was the Malay version of the pregnancy physical activity questionnaire (PPAQ) [36], which was originally developed by Chasan-Taber et al. [37]. Section D was used to calculate caloric intake among respondents in a day. The PPAQ section contained a total of 32 items pertaining to physical activities in five different activity domains, namely household, sports/exercise, commuting, occupational and inactivity.

### Study variables

The dependent variable in this study was physical inactivity. The independent variables were age, ethnicity, marital status, household income, education level, occupation, husband’s occupation, primigravida, grand multigravida, caloric intake per day, BMI at booking, medical problem in previous pregnancy and history of miscarriage. The operational definition of each variable used in this study is presented in Table 1.

**Table 1:** Operational definitions of variables

No	Word/Phrase	Operational Definition	Measurement Scale
1	Physical inactivity (Dependent variable)	Less than 7.5 METs^a^*hour/week or 150 min of moderate to vigorous activities in a week [1]	Yes/No
2	Age	Age at first antenatal booking based on identity card	Number in years
3	Marital status	Marital status verified by a legal marriage certificate	Married Unmarried
4	Primigravida	Pregnant for the first time	Yes/No
5	Ethnicity	Ethnicity as written in identity card	Malay Chinese Indian Others
6	Education level	Refers to the highest level of formal education	No formal education Primary school Secondary school Tertiary education
7	Household income	Combined household income according to the Department of Statistics Malaysia [38] categorized into low income (B40^b^), middle income (M40^c^) and high income (T20^d^)	B40^b^ < RM4850 M40^c^ RM4850-RM10959 T20^d^ ≥ RM10960
8	Caloric intake per day	Total energy intake in one day, measured by 24-h dietary recall (a proxy to hyperemesis gravidarum)	Numerical in Kilocalorie per day (kcal/day)
9	BMI^e^ at booking	Body mass index at booking at first trimester	Numerical in kg/m^2^
10	Occupation	Occupation reported by subject at booking	Public Private Self-employed Housewife/Unemployed
11	Husband’s occupation	Husband’s occupation reported by subject at booking	Public Private Self-employed Unemployed
12	Medical problem in previous pregnancy	Any medical problem or complications encountered during previous completed pregnancy (gestational diabetes, hypertensive disorders, anemia in pregnancy etc.)	Yes/No
13	History of miscarriage	Any experience of spontaneous pregnancy loss before completed 22 weeks of gestation	Yes/No
14	Grand multigravida	Pregnant for the sixth time or more	Yes/No

^a^ *METs* metabolic equivalents

^b^ B40 - bottom 40% earners in the population

^c^ M40 - middle 40% earners in the population

^d^ T20 - top 20% earners in the population

^e^ *BMI* body mass index

### Data analysis

Data were first analysed by using descriptive analysis. Continuous data were presented as the mean ± standard deviation (SD) or median and interquartile range (IQR) depending on the normality of the data distribution. Categorical data were presented as frequencies and percentages. Each of the respondent’s 24-h dietary recall was converted into total caloric intake per day by referring to the calorie bank developed by the Nutrition Department, Ministry of Health Malaysia, available on their website.

Physical activity levels were calculated from the answers given in the PPAQ, presented in METs*hour per week. It was also classified by intensity – sedentary (< 1.5 METs), light (1.5 to < 3 METs), moderate (3–6 METs) and vigorous (> 6 METs) – as well as by type/domain (household, sports, occupational, commuting and inactivity). The data were then classified as physically active or inactive to measure the prevalence of physical inactivity among the samples collected. Physical inactivity which was the dependent variable in this study was defined by less than 7.5 METs*hour per week which was equal to a total of at least 150 min of moderate to vigorous activities in a week [1].

The descriptive analysis was followed by simple and multiple logistic regressions to calculate the crude odds ratio and adjusted odds ratio (aOR) against the dependent variables in this study. In the multiple logistic regression analysis, all 13 independent variables included in this study were run and tested at once by using the ENTER method in SPSS version 22 to get the aOR and *p* value of each variable.

Significant factors associated with physical inactivity were confirmed when *p* < 0.05. This was followed by testing for interactions among the significant variables. Multicollinearity among the variables was also checked by looking at the standard errors of the significant variables in the final model. A value < 5.0 would confirm than there was no multicollinearity between the significant variables [39]. All analyses were done using SPSS software version 22.

## Results

**Fig. 2:**
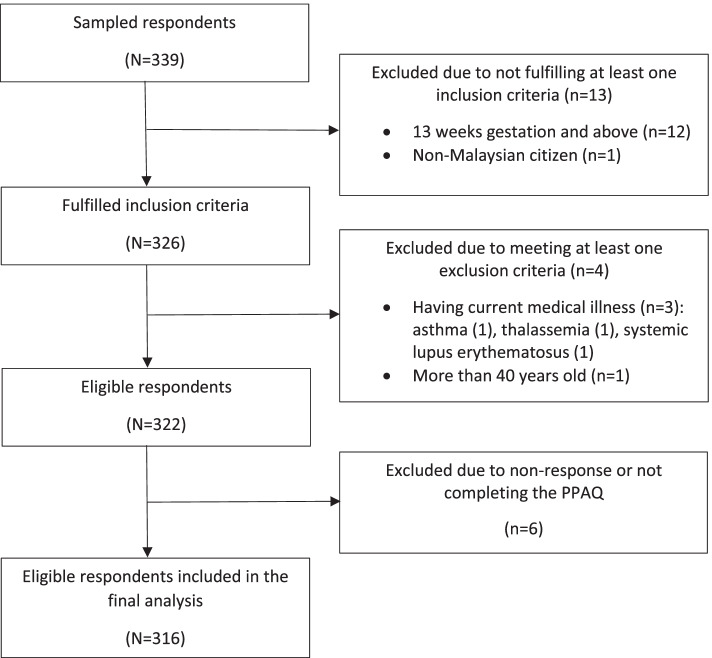
Respondents’ selection process

A total of 339 pregnant mothers were sampled and participated in this study. All those who were approached agreed to take part, however, 23 respondents were withdrawn from the study. This was because 13 respondents failed to meet at least one of the inclusion criteria and four respondents were found to have met at least one of the exclusion criteria. Another six respondents were withdrawn due to not completing the questionnaire properly, especially in PPAQ section. Therefore, a total of 316 respondents were included in the final analysis (Fig. 2). A total of 27.5% came from Cheras district, 26.6% from Titiwangsa district, 7.6% from Lembah Pantai district and 38.3% from Kepong district.

### Maternal characteristics and physical activity levels

The maternal characteristics included in this study are presented in Table 2. The mean age of respondents was 29 years old, and they were predominantly Malay. Most of the respondents were married and fell into the low-income category. Only 6 (2.1%) of them belonged to the high-income group (top 20% earners in the population); therefore, this group was merged with the middle-income group. For the education level variable, all respondents had formal education and only 2(0.6%) of the respondents fell under the primary education category. Therefore, the first three categories were merged, leaving only two categories in the final analysis, namely those who had had tertiary education (68.4%) and those who had not (31.6%). Few of the respondents had a history of miscarriage and medical illnesses in previous pregnancies such as gestational diabetes mellitus, anaemia in pregnancy or gestational hypertensive disorders.

**Table 2:** Maternal characteristics of respondents

Maternal characteristics	N (%)	Mean ± SD^**a**^
Age (year)	316(100.0)	29 ± 4.3
Ethnicity (*N* = 313)		
Malay	251 (80.2)	
Chinese	32 (10.2)	
Indian	19 (6.1)	
Others	11 (3.5)	
Occupation (*N* = 311)		
Public	60 (19.3)	
Private	157 (50.5)	
Self-employed	17 (5.4)	
Housewife/Unemployed	77 (24.8)	
Marital status (*N* = 310)		
Married	294 (94.8)	
Unmarried	16 (5.2)	
Education level (*N* = 316)		
No tertiary education^b^	100 (31.6)	
With tertiary education^c^	216 (68.4)	
Husband’s occupation (*N* = 308)		
Public	68 (22.1)	
Private	190 (61.7)	
Self-employed	47 (15.2)	
Unemployed	3 (1.0)	
Household income (*N* = 285)		
Low income (B40)	167 (58.6)	
Middle income (M40) and higher	118 (41.4)	
BMI^d^ at booking (kg/m^2^)	256(81.0)	24.7 ± 4.7
History of miscarriage (*N* = 314)		
Yes	17 (5.4)	
No	297 (94.6)	
Medical problem in previous pregnancy (*N* = 316)		
Yes	23 (7.3)	
No	293 (92.7)	
Grand multigravida (*N* = 316)		
Yes	11 (3.5)	
No	305 (96.5)	
Primigravida (*N* = 316)		
Yes	129 (40.8)	
No	187 (59.2)	
Caloric intake per day (kcal)	191(60.4)	1297 ± 350

^a^ *SD* standard deviation

^b^ formal education of 12 years or less

^c^ formal education of more than 12 years

^d^ *BMI* body mass index

Table 3 shows the total levels of physical activity among the pregnant mothers and further classified into domains and intensities of physical activity. The highest median activity was seen in the household domain – 92.5 METs*hour per week – followed by the occupational domain at 35.6 METs*hour per week. The lowest activity was detected in the sports/exercise domain. Besides, the pregnant mothers were also noted to spend more time doing light activities compared with moderate and vigorous activities. More than half of them met the WHO minimum physical activity requirement of engaging in at least 150 min of moderate to vigorous activities. The prevalence of physical inactivity among these respondents was 38.3%.

**Table 3:** Physical activity levels among respondents

Physical activity	N (%)	Median (IQR^**a**^)
Total activity (METs^b^*hour/week)	316(100. 0)	183.3 (188.8)
By domain (METs^b^*hour/week):		
Household activity	314(99.4)	92.5 (123.6)
Occupational activity	237(75.0)	35.6 (76.7)
Sports/Exercise activity	215(68.0)	0.9 (3.9)
Commuting activity	295(93.4)	10.7 (18.1)
Inactivity	313(99.1)	14.9 (24.7)
By intensity (METs^b^*hour/week):		
Sedentary activity	312(98.7)	7.3 (13.4)
Light activity	316(100. 0)	100.6 (100.0)
Moderate activity	305(96.5)	54.9 (100.2)
Vigorous activity	83(26.3)	0 (0.8)
Physically active^c^ (*N* = 316)		
Yes	195 (61.7)	
No	121 (38.3)	

^a^ *IQR* interquartile range

^b^ *METs* metabolic equivalents

^c^ Those who involved in at least 150 min of moderate to vigorous activities in a week

### Determinants of physical inactivity in pregnancy

In the simple logistic regression, education level, BMI at booking and primigravida were significantly associated with physical inactivity. These three variables were also significant in the multivariable analysis (Table 4).

**Table 4:** Simple and multiple logistic regressions to determine the factors associated with physical inactivity among pregnant mothers in Kuala Lumpur

Variables	Crude OR^**a**^	***p*** value	95% CI^**b**^	Adjusted OR^**a**^	***p*** value	95% CI^**b**^
Age (year)	0.97	0.19	0.92, 1.02	1.00	0.96	0.89, 1.13
Caloric intake per day (kcal)	1.00	0.26	1.00, 1.00	1.00	0.07	1.00, 1.00
Ethnicity						
Malay	1.00			1.00		
Chinese	1.02	0.96	0.48, 2.18	1.68	0.44	0.45, 6.24
Indian	1.89	0.18	0.74, 4.81	2.25	0.24	0.59, 8.58
Others	2.04	0.25	0.61, 6.87	1.36	0.81	0.12, 15.54
Grand multigravida						
Yes	2.88	0.18	0.61, 13.56	3.16	0.34	0.30, 33.87
No	1.00			1.00		
Primigravida						
Yes	1.70	0.02*	1.07, 2.70	3.54	0.01*	1.40, 8.97
No	1.00			1.00		
Occupation						
Public	0.94	0.85	0.47, 1.86	2.84	0.32	0.36, 22.38
Private	0.82	0.50	0.47, 1.44	1.33	0.58	0.48, 3.69
Self-employed	0.77	0.63	0.26, 2.29	3.03	0.09	0.85, 10.77
Housewife/Unemployed	1.00			1.00		
Marital status						
Married	1.00			1.00		
Unmarried	1.28	0.63	0.46, 3.54	0.42	0.50	0.03, 5.22
Education level						
No tertiary education^c^	1.70	0.03*	1.05, 2.76	3.77	0.01*	1.35, 10.52
With tertiary education^d^	1.00			1.00		
Husband’s occupation						
Public	1.00			1.00		
Private	0.91	0.73	0.51, 1.60	1.34	0.57	0.48, 3.72
Self-employed	1.03	0.93	0.48, 2.20	1.14	0.84	0.32, 4.04
Unemployed	3.04	0.37	0.26, 35.16	–	–	–
Household income						
Low income (B40^e^)	1.31	0.29	0.80, 2.14	0.96	0.93	0.40, 2.32
Middle income (M40^f^) or higher	1.00			1.00		
BMI^g^ at booking	0.92	0.01*	0.86, 0.97	0.88	0.01*	0.80, 0.97
History of miscarriage						
Yes	1.00			1.00		
No	2.08	0.21	0.15, 1.51	0.15	0.10	0.02, 1.45
Medical problem in previous pregnancy						
Yes	1.00			1.00		
No	1.83	0.22	0.70, 4.78	0.64	0.59	0.13, 3.17

^a^ *OR* odds ratio

^b^ *CI* confidence interval

^c^ formal education of 12 years or less

^d^ formal education of more than 12 years

^e^ B40 - bottom 40% earners in the population

^f^ M40 - middle 40% earners in the population

^g^ *BMI* body mass index

* Significant at *p* < 0.05

Nagelkerke R square 0.226

There were no interactions found between the three significant variables. The standard errors of all three significant variables were < 5.0 in the final model; therefore, it was safe to conclude that multicollinearity did not exist among these variables [39]. The Nagelkerke R square value was 0.226, which meant that the three significant variables found in this study would explain 22.6% of the variation in physical inactivity in the final adjusted model.

## Discussion

The observed prevalence of physical inactivity of 38.3% among pregnant women in this study is 10.0% higher compared with the general adult women population aged ≥16 years in Malaysia in 2019 [22]. This was expected because pregnant women tend to reduce and limit their physical activities during pregnancy [13, 15, 16, 24]. A possible explanation could be the belief that daily living activities provide sufficient exercise and that resting is preferred over being active due to the perceived health and safety risks to both mother and foetus while participating in sports and exercises [40]. Moreover, tiredness and pain have been reported as reasons for pregnant women to be inactive [24]. The prevalence of physical inactivity in this study, however, is much lower than the findings from another study in Malaysia, which was conducted in the neighbouring state of Negeri Sembilan. In that study, the prevalence of physical inactivity was as high as 60.4–71.9%, but the sample also comprised those in the second and third trimester of pregnancy, a factor that might explain the higher physical inactivity [21]. Another study conducted among pregnant women in Kuantan, Pahang, Malaysia, reported an even higher physical inactivity prevalence of 78.6% [41]. However, it was worth noting that the researchers used a different questionnaire, namely the Malay and short version of the International Physical Activity Questionnaire (IPAQ) instead of the PPAQ that was used in this study. The prevalence of physical inactivity was also noted to be higher among pregnant women in Brazil and Vietnam – 48.2 and 79.4%, respectively [15, 18]. It was particularly high in the study in Vietnam because the criterion applied to be physically active was 150 min per week of moderate to vigorous activities in the sports/exercise category only. In a study among pregnant women in Serbia, another middle-income country, the prevalence of physical inactivity was 11% lower than that reported in this study [20]. A study done in Ethiopia, a low-income country, reported an even lower physical inactivity prevalence of 21.9% [17]. These data are consistent with pooled worldwide data showing that the prevalence of physical inactivity is twice as high in higher compared with lower income countries [12].

The median of the total energy expenditure among pregnant women in this study is in the range of the reported mean/median total energy expenditure among pregnant women in previous studies from different countries, ranging from 123.2 METs*hour/week in Vietnam to 270.9 METs*hour/week in Portugal [16, 18, 27, 42–44]. At 183.3 METs*hour per week, the median total energy expenditure value in this study is almost the same as the findings among urban pregnant women in the state of Selangor, which surrounds the federal city of Kuala Lumpur geographically [45]. The pregnant women in our study spent most of their time doing moderate-intensity activities at home, even though most of them were working and only 24.8% of them were housewives or unemployed. This was evident because the household domain had the highest energy expenditure followed by occupational domain, whereas the sports/exercise domain had the lowest energy expenditure. Kaur et al. [45] found that the highest activity among urban pregnant women in Selangor was also in the household category. However, it was slightly lower at 76 METs*hour/week compared with the findings in this study. Moreover, studies in other parts of the world have also reported the highest activity in the household category [16, 18, 31, 46]. However, there were two studies which had found that occupational category had the highest energy expenditure in the first trimester but subsequently declined in the second trimester [27, 42]. Although high household activities among pregnant women have been a common theme in previous studies, it was worth investigating this category further because this study was conducted during the COVID-19 pandemic. Unforeseen circumstances like lockdowns and movement control orders imposed by the authorities of Malaysia – which resulted in new norms such as working from home and avoiding public spaces like gyms and antenatal classes, etc. – might be one of the explanations as to why this pattern emerged in this study. Furthermore, Kuala Lumpur was one of the states hit hardest by the COVID-19 outbreak in Malaysia [47]. This could well explain the low activity seen in the occupational and commuting category as most respondents spent the majority of their time at home, including working from home, instead of going out.

In this study, those who were primigravida were more likely to be physically inactive compared with those who were multigravida. Inexperienced first-time pregnant women might be extra cautious and mindful about their pregnancy and thus avoid sports and recreational activities, even when they have the time for these activities, compared with their counterparts who have to spend the bulk of their time caring for their living children. The concern for engaging in moderate and vigorous activities was evident in studies by Todorovic et al. [20] and Merkx et al. [14], in which women who were involved in light activities such as walking at a slow pace were more likely to continue this activity while those who used to be involved in moderate to vigorous activities were inclined to discontinue the practice entirely once they became pregnant.

Education level was another significant factor contributing to physical inactivity in this study in which, those who had no tertiary education (≤ 12 years of formal education) were more likely to be physically inactive. This is consistent with the findings by Lindqvist et al. [34] and also Todorovic et al. [20], in which those who had < 12 years of formal education were more likely to be physically inactive. Another study also found that illiteracy or no formal education was associated with higher physical inactivity [17]. Women with higher education could be more aware of the benefits of physical activity in pregnancy due to a greater ability to seek and process information given to them by health care providers. This finding emphasises the need to use simple language and terminology in physical activity educational programs as well as in promotional tools and instruments such as infographics, posters, leaflets and pamphlets, among others.

BMI at booking is a modifiable factor that was significantly associated with physical inactivity in this study. Those with lower BMI were more likely to be physically inactive. However, a previous study by Lindqvist et al. [34] reported a different result: those with a higher BMI were more likely to be inactive. Two other studies did not find BMI to be significantly associated with physical activity levels [15, 20], while Santo et al. [35] found that those who were underweight were more likely to be physically active compared with those with normal BMI. Further research is needed to extrapolate the inconsistencies with findings related to BMI.

There was no association between physical inactivity and maternal occupation found in this study as well as maternal age, although the NHMS 2019 reported a significant increase in physical inactivity level with age in the general adult population. However, that was only applied to the age group of 55–59 years to 75 years and above [22]. Past studies among pregnant women have also not seen any association with age [15, 19, 20, 30, 31, 33–35]. However Gebregziabher et al. [17] found that those who were less than 19 years of age were four times more likely to be physically inactive (aOR 4.7, 95% CI 1.4, 15.3) compared with those who were in 25–29-year age group. A study by Steinl et al. [32] among pregnant teenagers also noted a positive correlation between age and physical activity levels. However, this current study did not include teenage pregnancies because they are considered to be high risk.

It is also worth noting that those who had history of miscarriage or medical illness in previous pregnancies had no effect on physical activity levels in this study. This is in contrast to the findings by Gebregziabhe et al. [17], in which past miscarriage experience was found to be a barrier to being physically active in the current pregnancy. Therefore, it was worth studying whether healthy pregnant women with a history of miscarriage or medical illness perceive physical activity as highly associated with foetal and newborn morbidity and mortality when in fact it is highly unlikely, as has been reported by Davenport et al. [4].

### Strengths and limitations

There were only two studies that targeted the subpopulation of pregnant women in Malaysia between 1980 and 2019. This constituted only 0.6% of the total research on physical activity conducted in Malaysia within that period of time [48]. To the best of our knowledge, this was the first study assessing the physical inactivity levels among pregnant women in the most urbanised city of Kuala Lumpur.

This study used a specific questionnaire to measure physical activity levels in pregnancy, the Malay version of the PPAQ [36], which was first developed by Chasan-Taber et al. [37]. This questionnaire has been validated and translated into many languages and used around the world, making the comparison of findings of similar studies more standardised. However, using a self-administered questionnaire instead of an objective measurement tool such as accelerometer or pedometer could pose limitations such as information bias. Another limitation of this study is the convenience sampling used during data collection; hence, the results could not be generalised to the entire population of Kuala Lumpur. This was partly due to the unprecedented COVID-19 outbreak in the middle of the study, a factor that made it more difficult to continue sampling and collecting data using a stricter and more rigorous method.

## Conclusion

The prevalence of physical inactivity among pregnant women in their first trimester in Kuala Lumpur was 38.3%. The highest energy expenditure was seen in the household category, followed by the occupational category, whereas the sports/exercise category had the lowest energy expenditure, most likely due to these respondents spending their time isolating at home. There were little to no participation observed in the vigorous intensity category. Factors associated with physical inactivity were primigravida, BMI at booking and education level.

From the results of this study, it is recommended that health education and interventional programs on physical activity among pregnant women in their first trimester should be targeted and focused on those who are primigravida and have no tertiary education (≤ 12 years of formal education). The educational content and materials should be updated and tailored to the current COVID-19 pandemic situation in which self-isolation, working from home and social distancing are the new norms. This can be done by advocating for more home-based, moderate to vigorous intensity activities without using expensive pieces of exercising equipment.

## Data Availability

The datasets used and/or analysed during the current study are not publicly available in order to ensure participants’ privacy and confidentiality but are available from the corresponding author on reasonable request, with the permission from the Director General of Health, Malaysia.

## Abbreviations

aOR: Adjusted odds ratio
CI: Confidence interval
PPAQ: Pregnancy physical activity questionnaire
BMI: Body mass index
METs: Metabolic equivalents
MET: Metabolic equivalent of task
WHO: World Health Organization
MCH: Maternal and child health
SD: Standard deviation
IQR: Interquartile range
B40: Bottom 40% earners
M40: Middle 40% earners
T20: Top 20% earners
IPAQ: International physical activity questionnaire
